# A dark green fluorescent protein as an acceptor for measurement of Förster resonance energy transfer

**DOI:** 10.1038/srep15334

**Published:** 2015-10-15

**Authors:** Hideji Murakoshi, Akihiro C. E. Shibata, Yoshihisa Nakahata, Junichi Nabekura

**Affiliations:** 1 Supportive Center for Brain Research, National Institute for Physiological Sciences, Okazaki, Aichi 444-8585, Japan; 2 Department of Physiological Sciences, SOKENDAI (The Graduate University for Advanced Studies), Okazaki, Aichi 444-8585, Japan; 3 Precursory Research for Embryonic Science and Technology, Japan Science and Technology Agency (JST), Kawaguchi, Saitama 332-0012, Japan; 4 Division of Homeostatic Development, National Institute for Physiological Sciences, Okazaki, Aichi 444-8585, Japan; 5 Core Research for Evolutional Science and Technology, Japan Science and Technology Agency (JST), Kawaguchi, Saitama 332-0012, Japan

## Abstract

Measurement of Förster resonance energy transfer by fluorescence lifetime imaging microscopy (FLIM-FRET) is a powerful method for visualization of intracellular signaling activities such as protein-protein interactions and conformational changes of proteins. Here, we developed a dark green fluorescent protein (ShadowG) that can serve as an acceptor for FLIM-FRET. ShadowG is spectrally similar to monomeric enhanced green fluorescent protein (mEGFP) and has a 120-fold smaller quantum yield. When FRET from mEGFP to ShadowG was measured using an mEGFP-ShadowG tandem construct with 2-photon FLIM-FRET, we observed a strong FRET signal with low cell-to-cell variability. Furthermore, ShadowG was applied to a single-molecule FRET sensor to monitor a conformational change of CaMKII and of the light oxygen voltage (LOV) domain in HeLa cells. These sensors showed reduced cell-to-cell variability of both the basal fluorescence lifetime and response signal. In contrast to mCherry- or dark-YFP-based sensors, our sensor allowed for precise measurement of individual cell responses. When ShadowG was applied to a separate-type Ras FRET sensor, it showed a greater response signal than did the mCherry-based sensor. Furthermore, Ras activation and translocation of its effector ERK2 into the nucleus could be observed simultaneously. Thus, ShadowG is a promising FLIM-FRET acceptor.

The Förster resonance energy transfer (FRET) method allows to monitor protein-protein interactions, protein structural changes, and the activity of signaling proteins such as small GTPases and protein kinases^1^,^2^,^3^. An excellent method for monitoring FRET is fluorescence lifetime imaging microscopy (FLIM), which allows to quantitatively visualize FRET by measuring fluorescence lifetime changes of donor fluorophore^4^. The frequently used pair of fluorescent proteins for FLIM-FRET is EGFP as an energy donor and monomeric red (mRFP) or cherry (mCherry) fluorescent protein as an energy acceptor^5^,^6^,^7^. Because this pair has the advantage of well-separated emissions, it prevents spectral contamination, e.g., bleed-through from directory-excited mRFP/mCherry fluorescence to the channel for detection of EGFP fluorescence. Nonetheless, EGFP and mRFP/mCherry emission spectra occupy a wide range of wavelengths (500–650 nm), which makes it difficult to use additional fluorescent dyes or fluorescent proteins for dual observation with FLIM.

A low-quantum-yield version of a GFP mutant named RS8/ORG18 was created by random mutagenesis (at Y145, H148, V150, F165, and I167)^8^. On the basis of this finding, a dark yellow fluorescent protein (YFP) mutant called resonance energy-accepting chromoprotein (REACh) was developed and applied to FLIM^9^. Since the FRET pair of EGFP and REACh occupies only a narrow range of wavelengths for the measurement, this situation enables the dual observation with another fluorescent protein such as RFP^9^. More recently, the improved version of REACh called super REACh (sREACh) was developed by introducing maturation-improving mutations, and this protein successfully enhanced the FRET signal^10^. However, even under microscopically optimized conditions, REACh mutants emit weak fluorescence, which can produce unexpected artifacts due to contamination of the EGFP fluorescence with REACh/sREACh short-lifetime fluorescence (~0.32 ns)^9^, especially when hetero-molecules (which show a different local concentration) are paired for FRET. Therefore, a darker fluorescent protein that has high absorption and a low quantum yield is needed for reliable and sensitive FLIM-FRET.

We aimed to develop a dark sREACh with a greatly reduced quantum yield and good absorption properties. We engineered a very dark green fluorescent protein named ShadowG. When ShadowG was paired with mEGFP with the A206K mutation (in EGFP) and was applied to FRET sensors, we observed a robust FRET signal with reduced cell-to-cell variability of both the basal and response signal, most likely due to the superior folding and maturation properties of ShadowG.

## Results

**Figure 1:**
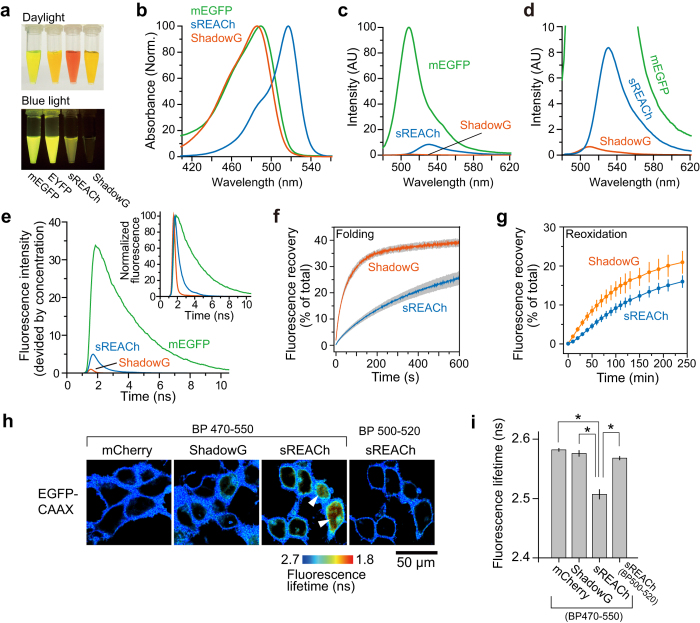
Spectrofluorimetric analysis of purified ShadowG. (**a**) Purified proteins (300 μM) under daylight (top) and blue LED light (bottom) are shown. (**b**) Normalized absorption spectra of mEGFP, sREACh, and ShadowG. (**c**) The emission spectra of mEGFP, sREACh, and ShadowG excited at 470 nm. For all samples, optical density at 470 nm is adjusted to the same value. (**d**) An enlarged view of panel c. (**e**) The fluorescence lifetime curves of the fluorescent proteins. 920 nm was used for 2-photon excitation. (**f**) Curves of refolding of sREACh and ShadowG from a denatured state. Three independent experiments were performed (the data are shown as mean ± SEM). (**g**) Fluorescence recovery of sREACh and ShadowG from a denatured/reduced state. Three independent experiments were performed (the data are shown as mean ± SEM). (**h**) A fluorescence lifetime image of HEK293 cells expressing EGFP-CAAX (a motif of K-Ras) which localizes on the plasma membrane. The cells also express cytosolic mCherry, ShadowG, or sREACh. The fluorescent images were taken with the indicated band pass (BP) filters. Arrow heads indicate bleed-through fluorescence of sREACh. The scale bar is 50 μm. (**i**) Quantification of (**h**). The fluorescence lifetime of cells expressing EGFP-CAAX along with mCherry, ShadowG, or sREACh was compared. A fluorescence lifetime decay curve averaged over the whole image were analyzed. The number of images is 10 for all conditions. Each image contains 3–8 cells. The data are presented as mean ± SEM. Asterisks denote statistical significance (p < 0.05, analysis of variance [ANOVA] followed by Scheffé’s *post hoc* test).

We aimed to develop a darker sREACh by error-prone PCR-based random mutagenesis. Mutations were introduced between amino acid positions Y143 and E235 (Supplementary Fig. S1); we avoided introducing any mutations into the chromophore. The PCR products with random mutations were then fused to the rest of the *sREACh* sequence (corresponding to amino acid residues 1–142) and ligated into a bacterial expression vector, constructing a genetic library. To screen the library for a dark sREACh mutant that has a high extinction coefficient but a low quantum yield, we first identified vividly colored colonies under day light, assuming that this characteristic is due to high absorption by the fluorescent protein in the bacteria. After that, the identified colonies were examined under blue light: we searched for dark colonies using a method similar to the one described elsewhere^11^. During the screening, we found a bright yellow colony that showed no fluorescence under blue light. Sequence analysis revealed 4 mutations (V148H, Y203T, A206K, R223F) in sREACh (Supplementary Fig. S1). These mutations are a combination of previously reported mutations^9^,^12^,^13^. The purified mutant has a yellow color under daylight and shows greatly reduced fluorescence under blue light (Fig. 1a). Spectral analysis confirmed an absorption peak at 486 nm and an emission peak at 510 nm (Fig. 1b–d and Table 1), similar to those of mEGFP. This blue-shifted spectrum of the sREACh mutant is most likely due to the Y203T mutation, which disrupts the π-stack^12^. Because this new mutant has very weak fluorescence and spectral properties similar to those of EGFP, we named this protein ShadowG where G stands for “green”. Further analysis revealed that the molar extinction coefficient of ShadowG is 89,000 M^−1^·cm^−1^, comparable to that of sREACh (Table 1). Quantum efficiency of ShadowG is 0.005, which is 14-fold smaller than that of sREACh (0.07; Table 1). Consistent with these results, the fluorescence lifetime of ShadowG (0.16 ns) is much shorter than that of sREACh (0.45 ns; Fig. 1e).

Next, to measure the folding kinetics, ShadowG was denatured in urea as described previously^14^, and the recovery of fluorescence was monitored after dilution in urea-free buffer. The fluorescence of denatured ShadowG recovered in 37s: faster than recovery of sREACh (308 seconds; Fig. 1f, Table 1). Next, chromophores of the urea-denatured ShadowG were reduced with dithionite, and reoxidation time and recovery were monitored after dilution in urea-free buffer. Reoxidation time of ShadowG (76 min) is half that of sREACh (133 min; Fig. 1g, Table 1).

Because ShadowG is darker than sREACh *in vitro*, we next tested the bleed-through effect of ShadowG in live cells. Cells were cotransfected with a plasmid encoding mCherry, ShadowG, or sREACh and a plasmid encoding mEGFP fused to the CAAX motif of K-Ras (Fig. 1h), and the fluorescence and its lifetime were monitored in the green channel with the band pass filter (letting wavelengths 470–550 nm through) under a 2-photon fluorescence lifetime imaging microscope (2pFLIM). Although mEGFP-CAAX was localized to the plasma membrane, mCherry, ShadowG, and sREACh were localized to the cytosol; therefore, the shorter lifetime of fluorescence of the cytosol was due to spectral contamination with these fluorescent proteins. Notably, when mEGFP-CAAX was coexpressed with sREACh, weak fluorescence with short lifetime was observed in the cytosol (Fig. 1h). This cytosolic fluorescence was removed by means of a narrow band pass filter (letting wavelengths 500–520 nm through), which blocks most of YFP fluorescence and a half of GFP fluorescence. This result suggests that cytosolic fluorescence with short lifetime was due to the spectral contamination with sREACh. The significant fluorescence contamination is not seen with mCherry and ShadowG. Quantification of fluorescence lifetime further confirmed that ShadowG yields significantly lesser fluorescence contamination (Fig. 1i).

**Figure 2:**
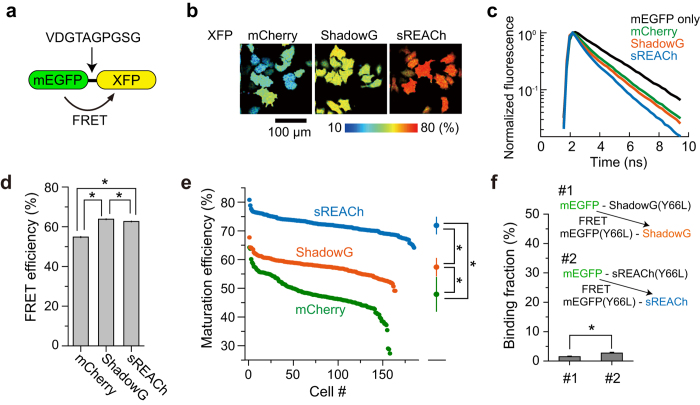
FRET efficiency and maturity of ShadowG. (**a**) A schematic drawing of the tandem fluorescent protein that we used to evaluate the FRET efficiency and chromophore maturation efficiency of ShadowG. XFP denotes mCherry, ShadowG, or sREACh. Amino acid sequence of the linker is shown. (**b**) Representative maturation efficiency images of the mEGFP-mCherry, mEGFP-ShadowG, and mEGFP-sREACh tandem mutants in HeLa cells. The scale bar is 100 μm. (**c**) Lifetime curves of mEGFP and mEGFP-XFPs. (**d**) Comparison of the FRET efficiency of the mEGFP-XFP tandems. We analyzed the fluorescence lifetime decay curve averaged over the whole image (See *Materials and Methods*). The number of images is 20 for all conditions. Each image contains 4–13 cells, and the data are presented as mean ± SEM. Asterisks denote statistical significance (p < 0.05, analysis of variance [ANOVA] followed by Scheffé’s *post hoc* test). (**e**) Maturation efficiency of individual cells was plotted in the descending order. The data are also presented as mean ± SD on the right. Asterisks denote statistical significance (p < 0.05, analysis of variance [ANOVA] followed by Scheffé’s *post hoc* test). (**f**) Inter-molecular FRET between tandem fluorescent proteins. To exclude the intra-molecular FRET, colorless mutation (Y66L) were introduced. Donor and acceptor (DNA molar ratio 1:1) were expressed in HeLa cells. The data are presented as mean ± SEM. Asterisks denote statistical significance (p < 0.05, *t* test).

Next, we characterized maturation efficiency and FRET efficiency of ShadowG when it was paired with mEGFP in live HeLa cells as described previously^10^. We expressed a tandem construct consisting of mEGFP and ShadowG, and the fluorescence lifetime of mEGFP was measured under the 2pFLIM (Fig. 2a–c). Since the fluorescence lifetime decay curves are convolution of both the FRET efficiency and maturity of acceptor, we measured these parameters separately for more detailed information, as described previously (Fig. 2d,e)^10^,^15^. The FRET efficiency of mEGFP-ShadowG was better than that of mEGFP-mCherry and mEGFP-sREACh, suggesting that mEGFP and ShadowG are a good FRET pair (Fig. 2d). When the maturation efficiency of ShadowG was compared with those of mCherry and sREACh, ShadowG showed higher maturation efficiency than mCherry but lower maturation efficiency in comparison with sREACh (Fig. 2e). Although, maturation efficiency is estimated by the fluorescence lifetime curve fitting using tandem fluorescent protein, there is a possibility that inter-molecular FRET between tandem fluorescent proteins due to the dimerization could affect this measurement, since fluorescent proteins tend to form dimer^13^. Therefore, to exclude this possibility, we measured the inter-molecular FRET between tandem fluorescent proteins, using Y66L chromophore-killed mutant^16^, and found that no significant dimer fraction exists in our experimental condition (Fig. 2f).

**Figure 3:**
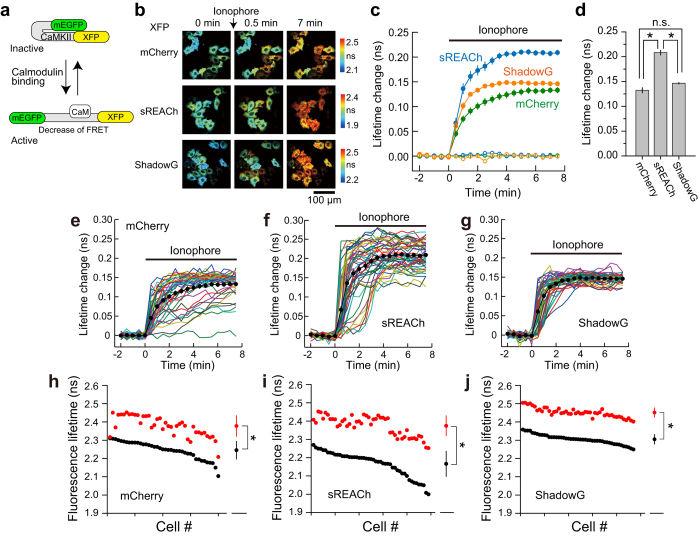
Performance of ShadowG in the configuration of Camuiα in HeLa cells. (**a**) A schematic of activation of the Camuiα mutant (CaMKII FRET sensor; see *Materials and Methods*). XFP denotes mCherry, sREACh, or ShadowG. (**b**) Representative fluorescence lifetime images of Camuiα coexpressed with calmodulin (DNA molar ratio 1:1) in HeLa cells after stimulation with the 10 μM ionophore 4-Bromo-A23187. Two-photon excitation at 920 nm was used for the excitation of mEGFP. The scale bar is 100 μm. (**c**) The averaged time course of fluorescence lifetime changes in response to application of the ionophore (closed circles) or dimethyl sulfoxide (DMSO; open circles). The number of cells analyzed is 40 for mCherry, 49 for sREACh, and 47 for ShadowG. In the DMSO experiment, the number of cells analyzed is 17 for mCherry, 31 for sREACh, and 24 for ShadowG. The data are presented as mean ± SEM. (**d**) The fluorescence lifetime changes (averaged over 6 to 7.5 min) after ionophore stimulation. The data are presented as mean ± SEM. Asterisks denote statistical significance (p < 0.05, analysis of variance [ANOVA] followed by Scheffé’s *post hoc* test). (**e,f**) The activation of Camuiα in individual HeLa cells after stimulation with the ionophore (the same data set as in panel c). Colored lines represent the response signals from individual cells, and the black circles indicate the averaged time course. The data are presented as mean ± SEM.(**h–j**) The basal fluorescence lifetime (averaged over −2 to 0 min) of individual cells is plotted in the descending order (black) along with the corresponding fluorescence lifetime (averaged over 6 to 7.5 min) after stimulation with the ionophore (red). The data from **(e**–**g)** are used in **(h**–**j)**, respectively. The data are also presented as mean ± SD on the right. Asterisks denote statistical significance (p < 0.05, *t* test).

To further test the performance of ShadowG, we compared the three single-molecule types of the CaMKII FRET sensor (Camuiα) design differing in their acceptor fluorophores (Fig. 3). mEGFP was fused to the N terminus, and mCherry, sREACh, and ShadowG were fused to the C terminus of full-length CaMKIIα: a configuration similar to the one previously reported^17^,^18^,^19^. The binding of Ca^2+^/calmodulin induced a structural change of CaMKII, leading to activation of CaMKIIα and a decrease in FRET (Fig. 3a). We transfected HeLa cells with one of the three CaMKII sensors and compared the response signals under the 2pFLIM^7^,^18^,^19^,^20^. After stimulation with a 10 μM ionophore, CaMKII was rapidly activated, and the signal reached a plateau within a few minutes (Fig. 3b–g). When the FRET response signal of mEGFP-CaMKII-ShadowG was compared with that of mCherry and sREACh, the signal was similar to that of mCherry and weaker than that of sREACh (Fig. 3d). Nonetheless, the response variability of individual cells was advantageously reduced with mEGFP-CaMKII-ShadowG than with either the mCherry or sREACh version (Fig. 3h–j). These data indicated that ShadowG-based Camuiα produces a stable response superior to that of the mCherry and sREACh versions.

**Figure 4:**
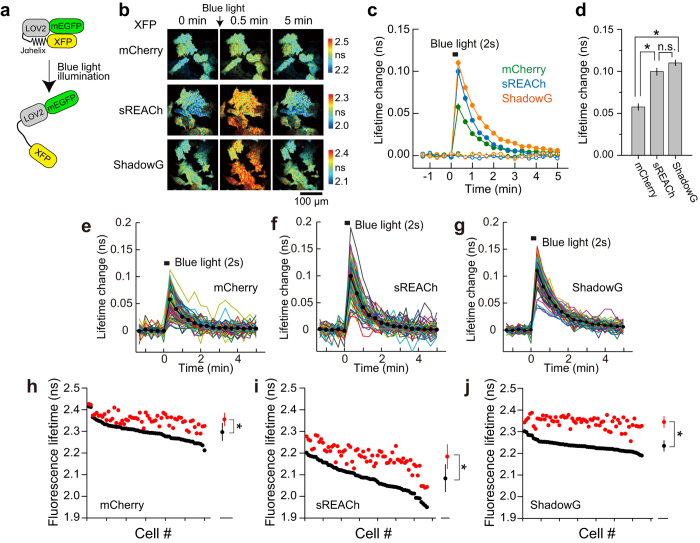
Performance of ShadowG in a FRET sensor based on the LOV2 in HeLa cells. (**a**) A schematic of a conformational change in the light-sensitive LOV2 domain. XFP denotes mCherry, sREACh, or ShadowG. (**b**) Representative fluorescence lifetime images of mEGFP-LOV2-XFP after illumination with blue LED light. Two-photon excitation at 920 nm was used for the excitation of mEGFP. The scale bar is 100 μm. (**c**) An averaged time course of fluorescence lifetime changes in response to illumination with blue light (closed circles) and without illumination (open circles). The number of cells analyzed is 60 for mCherry, 67 for sREACh, and 65 for ShadowG. In the no-light control, the number of cells analyzed is 26 for mCherry, 25 for sREACh, and 25 for ShadowG. The data are presented as mean ± SEM. (**d**) The fluorescence lifetime changes at 20 sec after blue light illumination. The data are presented as mean ± SEM. Asterisks denote statistical significance (p < 0.05, analysis of variance [ANOVA] followed by Scheffé’s *post hoc* test). (**e,f**) The conformational change of mEGFP-LOV2-XFP in individual HeLa cells after illumination with blue light (the same data set as in panel c). Colored lines represent the response signals from individual cells and the black circles indicate an averaged time course. The data are presented as mean ± SEM. (**h–j**) The basal fluorescence lifetime (averaged over −1.3 to 0 min) of individual cells is plotted in the descending order (black) along with the corresponding fluorescence lifetimes (at 20 sec) after blue light illumination (red). The data from **(e**–**g)** are used in **(h**–**j)**, respectively. The data are also presented as mean ± SD on the right. Asterisks denote statistical significance (p < 0.05, *t* test).

Next, we compared the three single-molecule types of LOV2 FRET constructs differing in their acceptor fluorophores (Fig. 4). The structure of the LOV2-Jα helix domain (which is a light-sensitive domain of Phototropin 1) is changed by absorption of blue light^21^. Namely, in the absence of blue light, it assumes a closed conformation, but after absorption of blue light, this domain reversibly opens^22^ (Fig. 4a). To evaluate the performance of ShadowG, we monitored FRET signals after the light-dependent structural changes in LOV2. mEGFP was fused to the N terminus of LOV2-Jα helix, and mCherry, sREACh, or ShadowG were fused to the C terminus, respectively. We transfected HeLa cells with one of the three LOV2 constructs and monitored the blue-light-dependent structural changes in the LOV2 FRET probes under the 2pFLIM (Fig. 4a–g). Decay was 40 s, consistent with another study^22^. When the FRET response signal of mEGFP-LOV2-ShadowG was compared with that of the mCherry and sREACh versions, the former signal was stronger than that of the mCherry construct and similar to that of the sREACh construct (Fig. 4d). As with Camuiα, the response variability of individual cells was reduced more strongly with mEGFP-LOV2-ShadowG than with the mCherry and sREACh versions (Fig. 4h–j). These data indicates that ShadowG-based LOV2 produces a stable response superior to that of the mCherry and sREACh versions. Furthermore, the reduced variability of ShadowG in comparison with sREACh was confirmed in HEK293 cells and dissociated neurons (Supplementary Fig. S2, S3).

**Figure 5:**
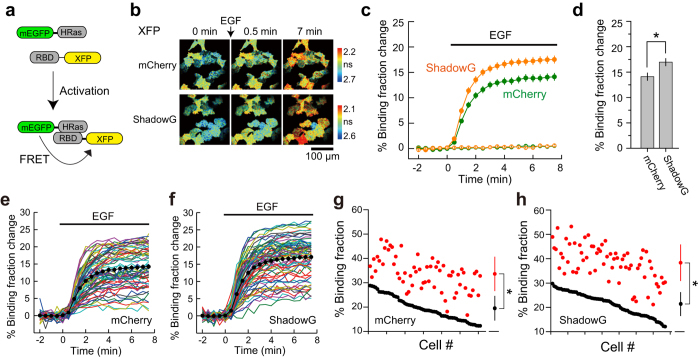
The performance of ShadowG in a FRET sensor of H-Ras activity in HeLa cells. (**a**) A schematic of the H-Ras activation. XFP denotes mCherry or ShadowG. (**b**) Representative fluorescence lifetime images of mEGFP-H-Ras in HeLa cells after stimulation with 50 nM EGF. Two-photon excitation at 920 nm was used for the excitation of mEGFP. The scale bar is 100 μm. (**c**) An averaged time course of fluorescence lifetime changes in response to stimulation with EGF (closed circles) or with empty buffer (open circles; control). Cells showing a 10–30% basal binding fraction (before stimulation) were chosen for the analysis. The number of cells analyzed is 66 for mCherry and 61 for ShadowG. In the control, the number of cells analyzed is 66 for mCherry and 68 for ShadowG. The data are presented as mean ± SEM. (**d**) The fluorescence lifetime changes (averaged over 6 to 7.5 min) after stimulation with EGF. The data are presented as mean ± SEM. Asterisks denote statistical significance (p < 0.05, *t* test). (**e**,**f**) The activation of H-Ras in individual HeLa cells after stimulation with EGF (the same data set as in panel c). Colored lines represent the response signals from individual cells, and the black circles indicate the averaged time course. The data are presented as mean ± SEM. (**g**,**h**) The basal fluorescence lifetime (averaged over −2 to 0 min) of individual cells is plotted in the descending order (black) along with the corresponding fluorescence lifetime (averaged over 6 to 7.5 min) after stimulation with EGF (red). The data from (**e**,**f**) are used in (**g**,**h**), respectively. The data are also presented as mean ± SD on the right. Asterisks denote statistical significance (p < 0.05, *t* test).

Next, ShadowG was applied to the previously reported separate-type H-Ras FRET sensor (FRas2-M)^23^, and was compared the FRET signal with that of mCherry (Fig. 5). We only compared with mCherry, but not with sREACh, since it has bleed-thorough effect (Fig. 1h, i). We used mEGFP-H-Ras and the Ras-binding domain (RBD)-ShadowG via the P2A sequence to ensure equal expression of these molecules^24^ and to minimize the response variability due to the imbalanced expression of the donor and acceptor. We transfected HeLa cells with this modified FRas2-M and compared the response signals under the 2pFLIM. After stimulation with epidermal growth factor (EGF), H-Ras was rapidly activated (within a few minutes; Fig. 5b,c,e,f). When the FRET response signal of Ras sensor with ShadowG was compared with that of mCherry, the former signal was stronger (Fig. 5d), suggesting that ShadowG is superior to mCherry as an acceptor. Reduced variability of basal FRET and the response signal—which was seen in the CaMKII and LOV2 sensor—was not observed (Fig. 5g, h).

**Figure 6:**
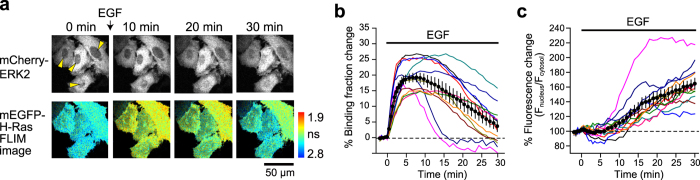
Dual observation of H-Ras activation and ERK2 translocation into nucleus. (**a**) Representative fluorescence lifetime images of mEGFP-H-Ras and mCherry-ERK2 fluorescence images after stimulation with 50 nM EGF. HeLa cells were transfected with RBD-ShadowG-P2A-mEGFP-H-Ras, mCherry-ERK2, and MEK1. We used 2-photon excitation (980 nm) of mEGFP and mCherry. Yellow arrowheads indicate the nuclei of individual cells. The scale bar is 50 μm. (**b**) Activation of H-Ras in individual HeLa cells after stimulation with EGF. Colored lines represent the response signals from individual cells, and the black circles indicate an averaged time course. The data are presented as mean ± SEM. (**c**) The time course of translocation of mCherry-ERK2 into the nucleus. The fluorescence intensity in the nucleus was divided by that in the cytosol and plotted in the graph. Colored lines represent the response signals from individual cells, and the black circles indicate the averaged time course. The data are presented as mean ± SEM.

By taking advantage of the narrow bandwidth of FRET in the pair mEGFP-ShadowG, we simultaneously imaged H-Ras activation and translocation of ERK into the nucleus after stimulation with EGF (Fig. 6). ERK is a well-known kinase which is phosphorylated by the Ras/Raf/MEK signaling cascade^25^. After phosphorylation, ERK translocates into the nucleus where it phosphorylates transcription factors. HeLa cells were transfected with Ras sensor, mCherry-ERK2, and MEK1 for retention of ERK2 in the nucleus^26^,^27^, and Ras activation and mCherry were monitored under the 2pFLIM and by means of 2-photon fluorescence microscopy, respectively (Fig. 6a). After stimulation with EGF, Ras was rapidly activated, and the signal decayed in 25 min (Fig. 6b). Delayed by the Ras activation, ERK2 showed slow translocation into the nucleus (Fig. 6c). These observations demonstrated that the mEGFP-ShadowG FRET pair enables the dual observation with mCherry.

## Discussion

In this work, we successfully developed a new fluorescent protein, ShadowG, as a FLIM-FRET acceptor for pairing with mEGFP. ShadowG is darker than the previously reported dark YFP mutants, such as sREACh^10^. The new protein reduces the risk of artifacts due to spectral contamination (Fig. 1) and has folding and maturation kinetics better than those of previously reported dark fluorescent proteins such as sREACh and Ultramarine^10^,^28^. Another advantage of ShadowG is its signal stability. When ShadowG was applied to a CaMKII or LOV2 FRET sensor, HeLa cells expressing these sensors showed smaller basal FRET variability in comparison with mCherry and sREACh constructs (Figs 3 and 4). Similar results were obtained with HEK293 and hippocampal neurons (Supplementary Fig. S2, S3). One simple explanation of the minimized variability is that sensor quality is improved due to the superior protein folding (Fig. 1f, g)^19^. Although maturation efficiency of ShadowG in tandem construct is smaller than that of sREACh (Fig. 2), the response signal of the ShadowG FRET sensor is comparable to that of the sREACh, depending on the sensor type (Fig. 4).

The contamination of the donor channel with acceptor fluorescence interferes with the acquisition of a correct FRET signal, especially with a hetero-FRET sensor that shows differential localization within the cell. However, since ShadowG has very low quantum efficiency, it could be applied to a hetero-type FRET sensor (Fig. 5) and used for dual observation with another signaling molecule. Taken together, these results show that ShadowG is a promising acceptor for precise multi-color FLIM-FRET.

## Materials and Methods

### Random mutagenesis

The *sREACh* gene was used as the initial template for construction of the genetic libraries. First, *XhoI* restriction site was silently introduced at the positions corresponding to amino acids L141 and E142 in *sREACh*, and the recombinant sequence was subcloned into the pRSET vector (Invitrogen). Random mutagenesis was performed by amplifying *sREACh* (the fragment corresponding to amino acid positions 141–238) by means of error-prone PCR using the Diversity PCR Random Mutagenesis Kit with a high error rate (7.2/1000 bp; Takara). Subsequently, the PCR fragments were digested with *Xho*I and *BsrG*I and ligated into the *sREACh*-containing pRSET vector. This construct was then introduced into electrocompetent cells, and the cells were grown for 18–20 h at 34 °C on LB agar plates supplemented with antibiotics.

### Plasmid construction

For construction of the EGFP-CAAX vector (used in Fig. 1h), the DNA sequence of the CAAX motif of K-Ras (corresponding to amino acid residues 173–188) was inserted into pEGFP-C1 (Clontech) with a linker encoding the peptide SGLRSRAQASNSAV. To create the cytosolic mCherry, sREACh, or ShadowG (used in Fig. 1h), we inserted the respective genes into the modified pEGFP-C1 vector, replacing *EGFP*. To create the tandem fluorescent protein constructs (used in Fig. 2), mCherry, sREACh, or ShadowG were inserted into multiple cloning site in the modified pEGFP-C1 vector, respectively. For construction of mEGFP-CaMKIIα-mCherry, mEGFP-CaMKIIα-sREACh, or mEGFP-CaMKIIα-ShadowG, mEGFP was fused to the N terminus of the full-length CaMKIIα sequence with the linker peptide SGLRSRA. Subsequently, *mCherry, sREACh*, or *ShadowG* was subcloned into the C-terminal region of *CaMKIIα* with a linker encoding the peptide GSNQQIFLRDIEQVPQQ. For construction of the mEGFP-LOV2-mCherry, mEGFP-LOV2-sREACh, or mEGFP-LOV2-ShadowG vector, we fused *mEGFP* (DNA sequence corresponding to amino acid residues 1–235) to the N terminus of LOV2 domain (DNA sequence corresponding to amino acid residues 404–546 in Phototropin1) with a linker encoding the peptide ASM. Then, mCherry, sREACh, or ShadowG was subcloned into the C-terminal region of LOV2 with the linker peptide KLGNS. For construction of the H-Ras FRET sensors, we fused mCherry, sREACh, or ShadowG to the RBD of Raf1 (amino acid residues 50–131) with F-Ras2-M mutations (K65E, K108A)^23^ and the linker peptide GSG. Subsequently, mEGFP-Ras with the linker peptide SGLRSRG was fused to the C terminus of the above protein via the P2A sequence^24^ so that the RBD and H-Ras parts were translated into different polypeptides within the cell.

For construction of mCherry-ERK2 and MEK1 plasmids, the source pCMV5-ratERK2-WT and EYFP-ERK2 plasmids were provided by Natalie Ahn (Addgene plasmid # 40812)^29^ and by Akihiko Yoshimura, respectively. *mCherry* and *ERK2* or *MEK1* was subcloned into the modified pmEGFP-C1, replacing *mEGFP*.

### Fluorescence properties of the fluorescent proteins

For bacterial expression of the His-tagged mEGFP, sREACh, or ShadowG, the respective genes were inserted into the pRSET vector (Invitrogen). His-tagged fluorescent proteins were overexpressed in *Escherichia coli* DH5α cells and purified on a Ni^+^-nitrilotriacetate column (HiTrap, GE Healthcare). Absorption and emission spectra of the fluorescent proteins that were diluted in PBS were measured on a UV-Vis spectrophotometer (UV-1800; Shimadzu) or a fluorescence spectrophotometer (RF-5300PC; Shimadzu), respectively. The molar concentration of the purified proteins was measured by the Bradford protein assay (Bio-Rad), considering the molecular weights predicted for the His-tagged fluorescent proteins. The molar extinction coefficients were determined by dividing the optical density of proteins by the molar concentration. The quantum efficiency (QY) of proteins was determined by comparison with QY (0.925) of fluorescein diluted in 0.1 M NaOH, as described previously^30^.

### Refolding and reoxidation kinetics

To analyze the refolding kinetics of sREACh and ShadowG, we denatured the proteins and refolded them as described previously^14^. Briefly, the purified proteins were dissolved in denaturation buffer (8 M urea, 1 mM dithiothreitol) and heated at 95 °C for 5 min for denaturation. We initiated the refolding reactions by diluting the denatured protein with a 100-fold volume of renaturation buffer (5 mM KCl, 2 mM MgCl_2_, 50 mM Tris pH 7.5, 1 mM dithiothreitol) at room temperature. In the reoxidation experiment, 5 mM dithionite was added to the denaturation buffer to reduce the chromophore. The fluorescence recovery was measured using a fluorescence spectrophotometer at 510 nm (RF-5300PC; Shimadzu).

### Cell culture and transfection

HeLa or HEK293 cells were cultured in Ham’s F12 medium (supplemented with 10% fetal bovine serum) at 37 °C in a humidified atmosphere containing 5% of CO_2_. Then, the cells were transfected with the plasmids using Lipofectamine 3000 (Invitrogen), followed by incubation for 12–24 h. Epifluorescence imaging and 2pFLIM-FRET imaging were performed in a solution containing 4-(2-hydroxyethyl)-1-piperazineethanesulfonic acid (HEPES; 30** **mM, pH 7.3)-buffered artificial cerebrospinal fluid (130** **mM NaCl, 2.5** **mM KCl, 1** **mM CaCl_2_, 1** **mM MgCl_2_, 1.25** **mM NaH_2_PO_4_, and 25** **mM glucose) at room temperature. The ionophore (4-Bromo-A23187) and EGF were purchased from Funakoshi Co., Ltd.

### Dissociated culture of hippocampal neurons and transfection

Cultured hippocampal neurons were prepared as described elsewhere^31^. Briefly, postnatal day 1 rats were anesthetized and decapitated, followed by brain removal and hippocampal tissue dissection. Neurons triturated by papain treatment were plated at a density of 1.3** × **10^4^ cells/cm^2^ on polyethyleneimine-coated 35** **mm culture dishes and maintained in serum-free Neurobasal medium supplemented with 2% B27, 2** **mM GlutaMAX-I, and 10** **mM HEPES at 37 °C and 5% CO_2_. After 11 days, neurons were transfected with 2 μg of either mEGFP-LOV2-sREACh or mEGFP-LOV2-ShadowG plasmid using Lipofectamine 2000 (Invitrogen) according to the manufacture’s protocol. After 24–48 hours, the neurons in the buffer (10** **mM HEPES, 150** **mM NaCl, 2.5** **mM KCl, 2** **mM CaCl_2_, 1** **mM MgCl_2_, 10** **mM glucose, and Tris-base for adjusting to pH 7.4) were imaged.

### Two-photon fluorescence lifetime imaging

Details of the 2pFLIM-FRET imaging were described previously^7^. Briefly, mEGFP in the FRET sensor was excited with a Ti-sapphire laser (Mai Tai; Spectra-Physics) tuned to 920 nm. The scanning mirror (6210H; Cambridge Technology) was controlled with a PCI board (PCI-6110; National Instruments) and ScanImage software^32^. The green fluorescence photon signals were collected by an objective lens (60×, 0.9 NA; Olympus) and a photomultiplier tube (H7422-40p; Hamamatsu) placed after a dichroic mirror (565DCLP; Chroma) and emission filter (FF01-510/84 or FF03-510/20 in Fig. 1h; Semrock). For dual observation of H-Ras activation and mCherry-ERK2 translocation, a Ti-sapphire laser tuned to 980 nm was used for simultaneous excitation of mEGFP and mCherry. The green fluorescence was acquired as described above, and mCherry’s fluorescent signal was collected by a photomultiplier tube (R3896; Hamamatsu) placed after the emission filter (FF01-625/90; Semrock). Measurement of fluorescence lifetime was carried out using a time-correlated single-photon counting board (SPC-150; Becker & Hickl) controlled with custom software^7^. For construction of a fluorescence lifetime image, the mean fluorescence lifetime in each pixel was translated into a color-coded image^33^. Analysis of the lifetime changes in individual cells was conducted as described previously^18^.

### Quantification of the FRET efficiency and mature fraction

To measure these parameters of the acceptor fluorescent protein in the tandem fluorescent protein in HeLa cells, we fitted the fluorescence lifetime curve to a double exponential function convolved with an instrument response function, *G*(*t*)^7^, assuming that two fractions exist in the cells: 1) mature mEGFP fused to an immature acceptor fluorescent protein where fluorescence lifetime of mEGFP (τ_mEGFP_) is 2.59** **ns; 2) mature mEGFP fused to a mature acceptor fluorescent protein where FRET occurs and fluorescence lifetime of mEGFP (τ_FRET_) gets shorter:
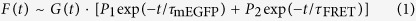
where *P*_1_ and *P*_2_ are the populations of mEGFP fused to the mature acceptor and immature acceptor, respectively. The mean FRET efficiency (*Y*_FRET_) between mEGFP and the mature acceptor was calculated as follows:
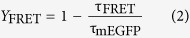
and the fraction of the mature acceptor (*A*_*mature*_) was calculated using the following formula:
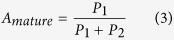


## Additional Information

**How to cite this article**: Murakoshi, H. *et al*. A dark green fluorescent protein as an acceptor for measurement of Förster resonance energy transfer. *Sci. Rep.* **5**, 15334; doi: 10.1038/srep15334 (2015).

## Supplementary Material

Supplementary Information

## Figures and Tables

**Table 1:** Comparison of the characteristics of ShadowG, mEGFP, and sREACh.

**Protein**	**EC (M**^**−1**^**cm**^**−1**^)	**QY**	**Abs (nm)**	**Em (nm)**	**Relative brightness**	**Folding t**_**1/2**_ **(sec)**	**Oxidation t**_**1/2**_ **(min)**	**Förster distance with mEGFP (nm)**
mEGFP	58000	0.73	488	507	100.0	—	—	—
	56000*	0.60*	488*	507*	—	—	—	—
sREACh	115000	0.07	517	531	19.0	308	133	5.8
ShadowG	89000	0.005	486	510	1.1	37	76	4.7

The quantum efficiency (QY) of fluorescent proteins was obtained using fluorescein in 1 M NaOH (0.925) as a reference. EC: extinction coefficient, t_1/2_: half-time of fluorescence recovery, Abs: absorption maximum, E_m_: Emission maximum.

The ECs were measured by means of the concentration of the chromophore determined by the alkaline denaturation method^34^.

^*^Values obtained from another study^35^.
