# Corrigendum: A dark green fluorescent protein as an acceptor for measurement of Förster resonance energy transfer

**DOI:** 10.1038/srep46840

**Published:** 2017-06-07

**Authors:** Hideji Murakoshi, Akihiro C. E. Shibata, Yoshihisa Nakahata, Junichi Nabekura

Scientific Reports 5: Article number: 1533410.1038/srep15334; published online: 10 15 2015; updated: 06 07 2017

This Article contains typographical errors.

In Table 1 under ‘Förster distance with mEGFP (nm)’, the sREACh and ShadowG values should read 6.2 and 5.1 respectively.

In addition, in the Materials and Methods section under subheading ‘Plasmid construction’,

‘For construction of mCherry-ERK2 and MEK1 plasmids, the source pCMV5-ratERK2-WT and EYFP-ERK2 plasmids were provided by Natalie Ahn (Addgene plasmid # 40812)^29^ and by Akihiko Yoshimura, respectively. *mCherry* and *ERK2* or *MEK1* was subcloned into the modified pmEGFP-C1, replacing *mEGFP*’.

should read:

‘For construction of mCherry-ERK2 and MEK1 plasmids, the source pCMV5-ratERK2-WT and EYFP-MEK1 plasmids were provided by Natalie Ahn (Addgene plasmid # 40812)^29^ and by Akihiko Yoshimura, respectively. *mCherry* and *ERK2* or *MEK1* was subcloned into the modified pmEGFP-C1, replacing *mEGFP*’.

