# Supplementary material for: A dark green fluorescent protein as an acceptor for measurement of Förster resonance energy transfer
Source: Sci Rep. 2015 Oct 15;5:15334. doi: 10.1038/srep15334 (PMC4606784; doi:10.1038/srep15334)
Supplement: Supplementary Information [file srep15334-s1.pdf]

Supplementary information

A dark green fluorescent protein as an acceptor for measurement of Förster resonance energy transfer

Hideji Murakoshi[1,2,3,]*, Akihiro C. E. Shibata[1], Yoshihisa Nakahata[4], and Junichi Nabekura[2,4,5]

[1] Supportive Center for Brain Research, National Institute for Physiological Sciences, Okazaki, Aichi 444-8585, Japan, [2] Department of Physiological Sciences, SOKENDAI (The Graduate University for Advanced Studies), Okazaki, Aichi 444-8585, Japan, [3] Precursory Research for Embryonic Science and Technology, Japan Science and Technology Agency (JST), Kawaguchi, Saitama 332-0012, Japan, [4] Division of Homeostatic Development, National Institute for Physiological Sciences, Okazaki, Aichi 444-8585, Japan, [5] Core Research for Evolutional Science and Technology, Japan Science and Technology Agency (JST), Kawaguchi, Saitama 332-0012, Japan

*Correspondence author: Hideji Murakoshi; e-mail: murakosh@nips.ac.jp; Tel.: +81 564-55-7857; Fax: +81 564-55-7858

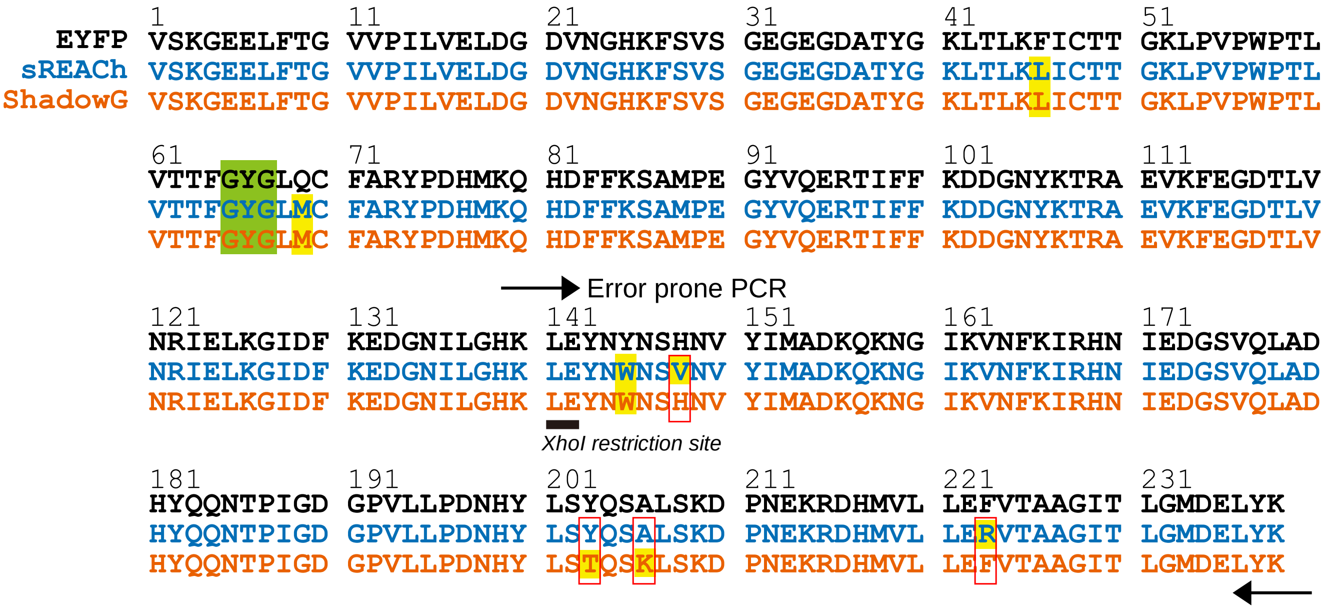

Fig. S1. Comparison of the amino acid sequence of EYFP, sREACh, and ShadowG. Yellow boxes indicate the differences between EYFP and sREACh or ShadowG. Red rectangles indicate the differences between ShadowG and sREACh. The chromophore tripeptide is highlighted with the green box.

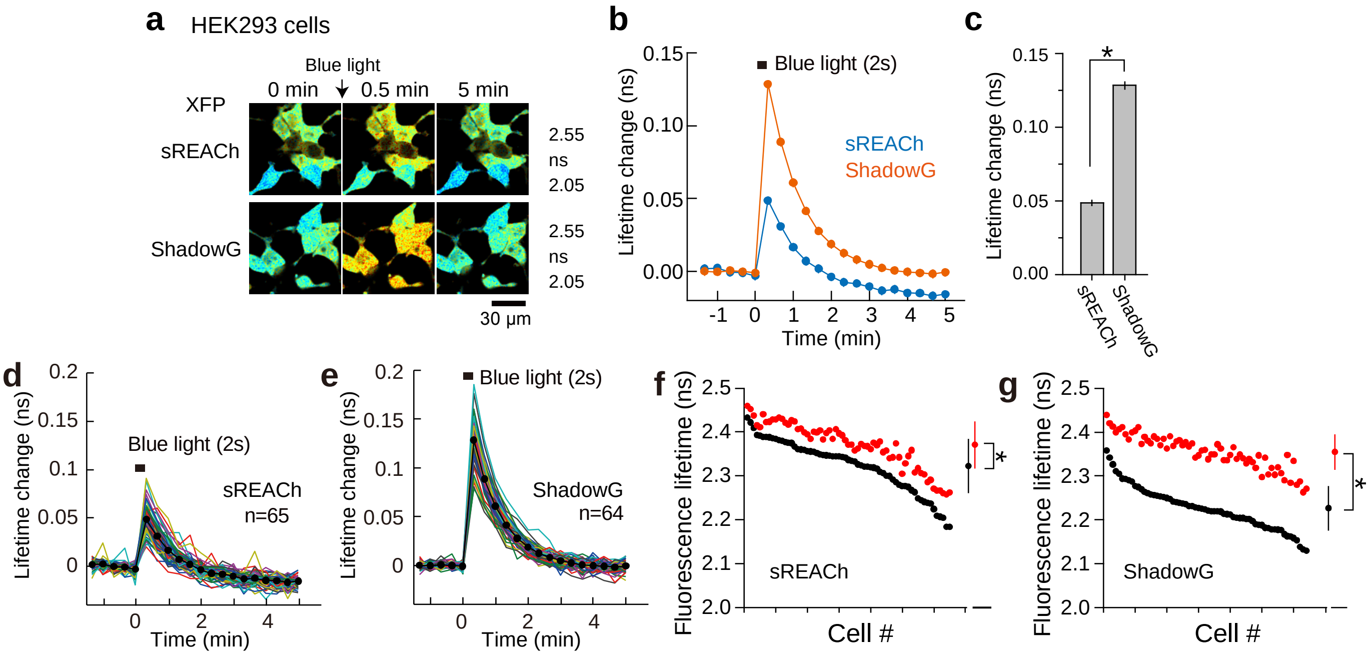

Fig. S2. Performance of ShadowG in a LOV2 FRET sensor in HEK293 cells.

(a) Representative fluorescence lifetime images of mEGFP-LOV2-XFP after illumination with blue LED light. XFP denotes sREACh or ShadowG. Two-photon excitation at 920 nm was used for the excitation of mEGFP. The scale bar is 30 µm.

(b) An averaged time course of fluorescence lifetime changes in response to illumination with blue light. The number of cells analyzed is 65 for sREACh and 64 for ShadowG. The data are presented as mean ± SEM.

(c) The fluorescence lifetime changes at 20 sec after blue light illumination. The data are presented as mean ± SEM. Asterisks denote statistical significance ($p < 0.05$, *t* test).

(d, e) The conformational change of mEGFP-LOV2-XFP in individual HEK293 cells after illumination with blue light (the same data set as in panel b). Colored lines represent the response signals from individual cells and the black circles indicate an averaged time course. The data are presented as mean ± SEM.

(f, g) The basal fluorescence lifetime (averaged over –1.3 to 0 min) of individual cells is plotted in the descending order (black) along with the corresponding fluorescence lifetimes (at 20 sec) after blue light illumination (red). The data from **(d)** and **(e)** are used in **(f)** and **(g)**, respectively. The data are also presented as mean ± SD on the right. Asterisks denote statistical significance ($p < 0.05$, *t* test).

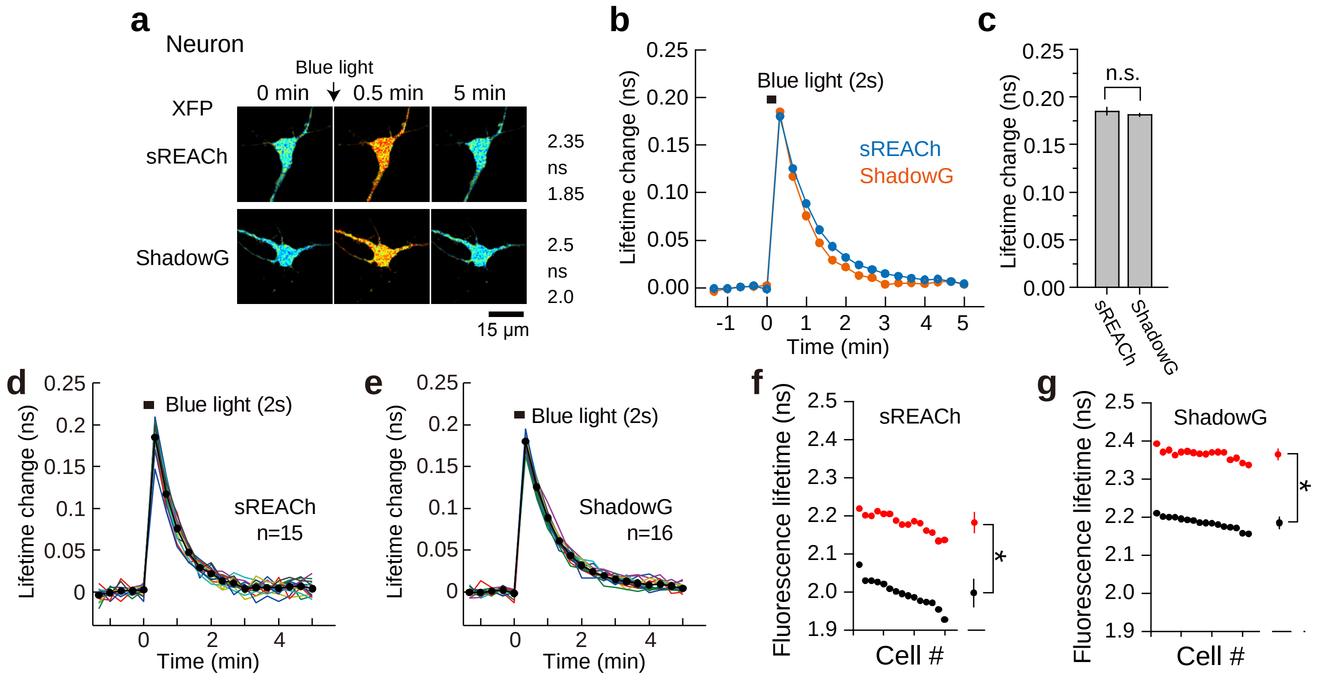

Fig. S3. Performance of ShadowG in a LOV2 FRET sensor in dissociated hippocampal neurons.

(a) Representative fluorescence lifetime images of mEGFP-LOV2-XFP after illumination with blue LED light. XFP denotes sREACh or ShadowG. Two-photon excitation at 920 nm was used for the excitation of mEGFP. The scale bar is 15 µm.

(b) An averaged time course of fluorescence lifetime changes in response to illumination with blue light. The number of cells analyzed is 15 for sREACh and 16 for ShadowG. The data are presented as mean ± SEM.

(c) The fluorescence lifetime changes at 20 sec after blue light illumination. The data are presented as mean ± SEM. There is no significant difference between sREACh and ShadowG ($p > 0.05$, *t* test, n.s., not significant).

(d, e) The conformational change of mEGFP-LOV2-XFP in individual neurons after illumination with blue light (the same data set as in panel b). Colored lines represent the response signals from individual cells and the black circles indicate an averaged time course. The data are presented as mean ± SEM.

(f, g) The basal fluorescence lifetime (averaged over –1.3 to 0 min) of individual cells is plotted in the descending order (black) along with the corresponding fluorescence lifetimes (at 20 sec) after blue light illumination (red). The data from **(d)** and **(e)** are used in **(f)** and **(g)**, respectively. The data are also presented as mean ± SD on the right. Asterisks denote statistical significance ($p < 0.05$, *t* test).
